# Initial management of septic complications in adult oncologic patients in the emergency department: A systematic review and meta-analysis

**DOI:** 10.1097/MD.0000000000043804

**Published:** 2025-11-28

**Authors:** Victor Melgar-Perez, Victor Serrano-Fernandez, Juan Manuel Carmona-Torres, Carmen Bouzas-Mosquera, Joseba Aingerun Rabanales-Sotos, Jose Alberto Laredo-Aguilera

**Affiliations:** a Hospital Universitario de Toledo, Toledo, Spain; b Facultad de Fisioterapia y Enfermeria, Universidad de Castilla-La Mancha, Toledo, Spain; c Grupo de Investigacion Multidisciplinar en Cuidados (IMCU), Universidad de Castilla-La Mancha, Toledo, Spain; d Facultad de Enfermería, Universidad de Castilla-La Mancha, Albacete, Spain; e Grupo de Actividades Preventivas en el ámbito Universitario de Ciencias de la Salud (GAP-CS), Universidad de Castilla-La Mancha, Albacete, Spain.

**Keywords:** emergency department, meta-analysis, oncology patients, sepsis, systematic review

## Abstract

**Background and aims:**

Cancer encompasses a group of diseases that progressively affect an increasing number of individuals. This condition elevates the risk of developing sepsis in these patients, requiring care in the emergency department. Therefore, this review aims to evaluate whether the initial management of septic complications in emergency departments adheres to current recommendations and enhances patient survival.

**Methods:**

Following the Preferred Reporting Items for Systematic Review and Meta-Analysis guidelines, a systematic review with meta-analysis was conducted using searches in PubMed, Scopus, Web of Science and BVS Library databases. Studies evaluating oncology patients with sepsis in the emergency department were selected. Additionally, a random-effects meta-analysis was performed assessing time to antibiotics due to the implementation of protocols. The Joanna Briggs Institute tools were used to assess the risk of bias for each type of study included.

**Results:**

The searches yielded 1040 results. After removing duplicates and filtering by title and abstract, 16 studies were selected for qualitative synthesis and 5 studies were selected for quantitative synthesis. Overall, the times from arrival at the emergency department to initial care did not meet established standards, which were associated with higher mortality rates. However, meta-analysis showed a reduction of 10.16 minutes on time to antibiotics due to the implementation of protocols. The most common infection sites were the urinary, digestive and respiratory tracts, with the latter 2 presenting the highest mortality rates. However, the use of scales and the implementation of specialized clinical staff help mitigate these problems.

**Conclusion:**

The management of septic complications does not comply with current regulations, highlighting the need for systematic protocols to improve care times. Implementation of protocols and early detection of complications through standardized scales and advanced clinical staff is essential to optimize care. Further research focused on these patients is needed to evaluate the effectiveness of interventions guided by expert personnel and specific scales.

## 1. Introduction

Cancer consists of a group of pathologies that progressively affect an increasing number of individuals.^[1]^ According to data from the International Agency for Research on Cancer, in 2040, there will be approximately 30 million new annual cases compared to 18 million diagnosed in 2018.^[1]^ In addition, a 54.9% increase is expected compared to 2020, specifically in males (60.6% vs 48.8% in females).^[2]^ The risk factors for developing sepsis can be categorized into 1) those that increase the risk of an infection leading to organ dysfunction, and 2) those that contribute to the occurrence of a primary infection.^[3]^ Among the latter, chronic diseases are the most frequent, with cancer being one of the most significant.^[3]^ Therefore, early detection and management of infections in oncology patients (OP) reduce the risk of septic complications and improves prognosis.^[4–6]^

Survival rates have increased owing to scientific advances.^[7]^ However, these patients often suffer permanent sequelae due to the underlying disease and the treatments used.^[3,8]^ One of the most important sequelae is immunosuppression in the form of neutropenia.^[7,8]^ Despite the decrease in mortality in recent years, the presence of any type of oncological process in septic patients significantly increases mortality (27.9% vs 19.5%),^[9]^ especially in young patients.^[7,10]^ A recent meta-analysis showed that two-thirds of OP with sepsis died.^[11]^ Additionally, mortality is 10 times higher in OP with sepsis compared to non-oncological population.^[12,13]^ This is mainly due to the late detection of infections.^[5,6,14]^

Due to the immunosuppression of OP, certain severe infections may be presented as afebrile or with only mild fever.^[7,8]^ In addition, when presenting to the emergency department (ED), one-third of patients with neutropenia and active infectious processes are afebrile, which has been associated with a worse prognosis due to delayed diagnosis and intervention.^[6,14,15]^ Infections are frequent and represent the primary cause of in-hospital morbidity in OP.^[4,7,16]^ Among patients with hematological tumors (HT) and solid tumors (ST), infectious processes account for the deaths of 60% and 50% of patients, respectively.^[4]^ In fact, 1 in 3 in-hospital deaths in the United States could be attributed to sepsis, while in Europe, sepsis-related death rates reach similar levels of 32.5%.^[15,17]^

Among OP, septic processes are the leading cause of admission to Intensive Care Units (ICU).^[18,19]^ Furthermore, 1 in 5 patients admitted to the ICU suffer from some type of cancer.^[10,11,13,16]^ Within ICU patients, 71% of those with HT and 41.5% with ST present sepsis, compared to 35.9% of non-oncological patients.^[9]^ These rates are common for OP with both active and inactive processes (12.6% vs 3.81%).^[8,15]^ The most frequent site of infection is the lung,^[10,13,14,16,20]^ with pneumonias responsible for 10% of admissions and 50% of septic shocks in OP.^[7]^ However, the highest mortality rates are associated with sepsis of intestinal origin, while the lowest are in those of genitourinary origin.^[16]^ Symptoms can vary depending on the site of infection, but generally include hemodynamic alterations, changes in urination, altered levels of consciousness, or changes in respiratory patterns.^[14,15]^

Currently, there is no single test that can diagnose sepsis independently, requiring non-immediate laboratory tests such as serum lactate, C-reactive protein (CRP) and procalcitonin determination.^[4]^ Additionally, scales like the Sequential Organ Failure Assessment (SOFA) score are designed to assess a patient’s condition, although they also require non-immediate laboratory tests.^[21]^ To address the need for early sepsis management in the ED, the quick Sequential Organ Failure Assessment (qSOFA) has been developed, allowing for an immediate initial assessment of the patient’s condition.^[14,21]^ The Third International Consensus Definitions for Sepsis and Septic Shock (Sepsis-3) recommends using this tool due to its utility in quickly and effectively detecting patients with sepsis.^[22]^ Another similar scale is the National Early Warning Score (NEWS-2), developed in the United Kingdom.^[23]^ On the other hand, the Multinational Association for Supportive Care in Cancer (MASCC) is internationally recommended for stratifying and triaging patients with neutropenia based on the degree of severity.^[14,24]^ However, these scales have certain limitations as they cannot be treated as definitive diagnostic tests on their own, but rather as evaluation guides.^[20,21,25]^ The integration of clinical information observed through these scales and the measurement of laboratory biomarkers could potentially improve the diagnosis and prognosis of sepsis in OP.^[4]^

Regarding the early initiation of antibiotic therapy, numerous articles and societies including the Infectious Diseases Society of America (IDSA), European Society of Emergency Medicine (EUSEM), and the National Institute for Health and Care Excellence (NICE) emphasize the importance of starting antibiotic treatment in OP within the first hour of presentation in the ED.^[5,6,20,22,25–28]^ A delay treatment beyond 1 hour has been shown to increase mortality by 8% each hour.^[6,25,27]^ OP presenting to the ED with suspected infection and/or sepsis should be triaged and treated withing the first 15 minutes.^[6,24]^

Given the controversy over the use of predictive scales,^[4,14,20–22,24,25,27–30]^ the implementation of protocols and specialists to improve prognosis,^[4,22,26–28]^ and doubts regarding the use of antibiotics,^[6,12,14,15,20,24–31]^ there is a need for a systematic review to study current trends in the initial care and mortality of OP in the ED.

Therefore, the aim of this review is to analyze whether the initial management of septic complications in the ED meets current recommendations and contributes to increasing survival in OP.

## 2. Methodology

### 2.1. Design and information sources

**Figure 1.**
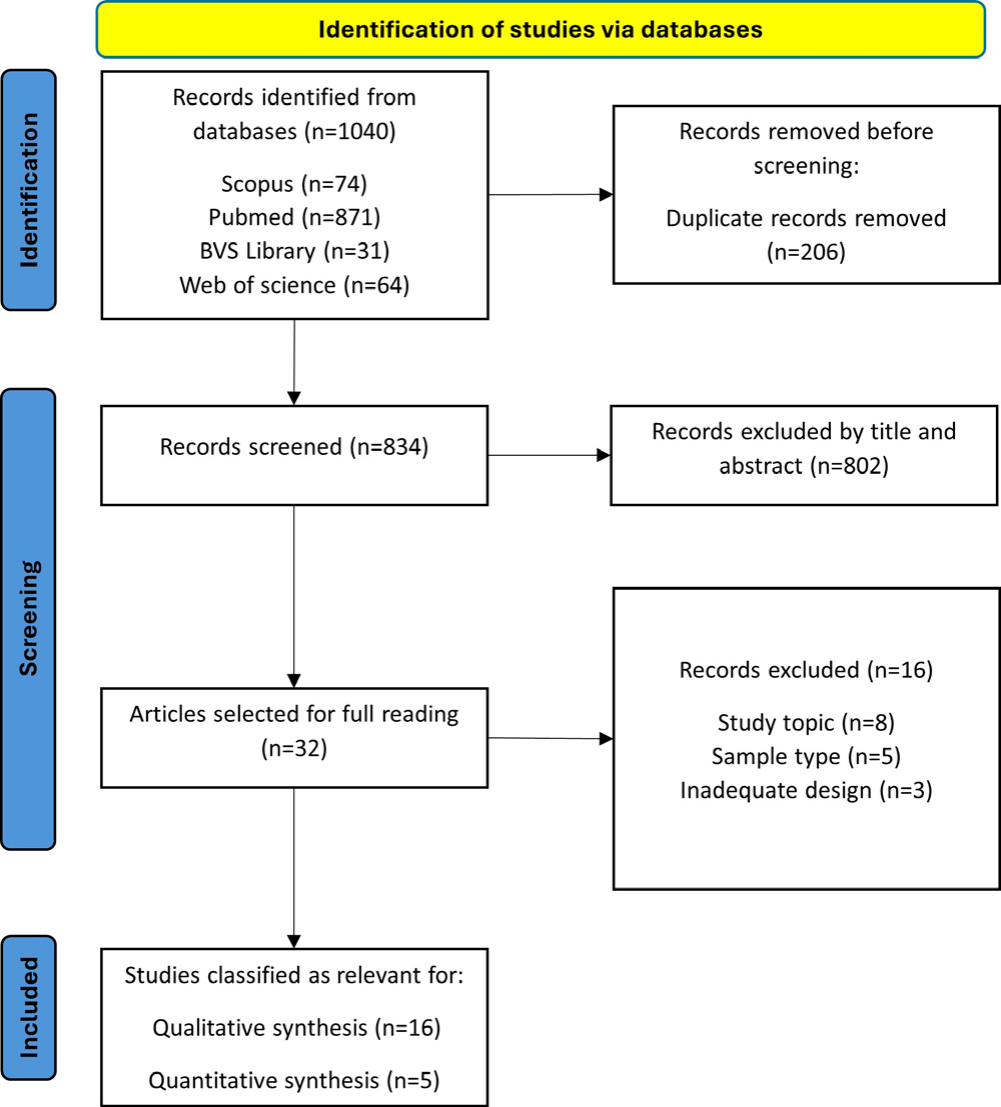
PRISMA flow diagram. PRISMA = preferred reporting items for systematic reviews and meta-analyses.

This study is a systematic review with meta-analysis conducted in accordance with the PRISMA (Preferred Reporting Items for Systematic Reviews and Meta-Analyses) guidelines.^[32]^ This review is registered in PROSPERO under the registration number CRD42024578521. The details of the study selection process are illustrated in Figure 1.

For information retrieval, we consulted the PubMed, Web of Science, Scopus and BVS Library databases.

### 2.2. Search strategy

The searches were designed to address the population, intervention, control, and outcome (PICO) question, which is outlined in Table 1. The searches were conducted in January and February 2025.

**Table 1:** PICO question.

Population	Intervention	Control	Outcome
Oncology adult patients	Initial management of septic complications in the ED following current recommendations	Selection of antibiotic treatment, use of scales and implementation of protocols	Meets recommendations and increases survival

ED = emergency department, PICO = population, intervention, control, and outcome.

The clinical question posed was: Does the initial management of septic complications in the ED meet current recommendations and increase survival in adult OP?

To answer this question, we developed a search strategy using Boolean operators (AND, OR and NOT) in combination with the MeSH terms listed in Table 2.

**Table 2:** Search string used in each database.

Database	Search string
Web of Science	(((((TS=(“sepsis”)) OR TS=(“septic shock”)) AND TS=(“oncology patient”)) OR TS=(“cancer patient”)) AND TS=(“emergency department”)) NOT TS=(“pediatric”)
BVS Library	(sepsis) OR (septic shock) OR (bloodstream infection) AND (oncology patient) OR (cancer patient) AND (emergencies) AND NOT (pediatric)
PubMed	(sepsis OR septic shock) AND (oncology patient OR cancer patient) AND (emergency department) NOT (pediatric)
Scopus	TITLE-ABS-KEY(“sepsis”) OR TITLE-ABS-KEY(“septic shock”) AND TITLE-ABS-KEY(“oncology patient”) OR TITLE-ABS-KEY(“cancer patient”) AND TITLE-ABS-KEY(“emergency department”) AND NOT TITLE-ABS-KEY(“pediatric”)

### 2.3. Inclusion and exclusion criteria

The studies included in this review were selected based on the following criteria: 1) studies with a sample of OP with sepsis; 2) studies conducted in the hospital ED; and 3) articles written in English or Spanish.

Conversely, articles with the following characteristics were excluded: 1) studies including pediatric patients in their sample; 2) studies involving non-oncological population; 3) studies including pediatric sample; 4) studies where the intervention was conducted outside the ED; 5) narrative reviews, systematic reviews and gray literature.

### 2.4. Selection process

The selection of studies for both qualitative and quantitative synthesis was carried out by 2 researchers (VMP, VSF) based on the described inclusion and exclusion criteria. In cases of doubt or discrepancy, the opinion of JALA was sought until a consensus was reached. The study selection process is depicted in the PRISMA flow diagram (Fig. 1).

Following the database searches, duplicates were removed using the Mendeley reference manager. Titles and abstracts were then reviewed to determine which studies would undergo full-text reading. After reviewing the full texts, studies that met the review objectives were considered.

### 2.5. Quality assessment

To evaluate the quality of the selected studies and detect risk of bias, the tools from the Joanna Briggs Institute (JBI) were used.^[33]^ These tools consist of several checklists depending on the type of study being evaluated. Each article was assessed according to its design using the corresponding scale, with each item answered as “Yes,” “No,” “Not applicable” or “Unclear.” A score of 1 was given to each fulfilled item and 0 for those that were not. The final score was the sum of each item.

Specifically, the quality of cohort and quasi-experimental studies was evaluated (Tables S1 and S2, Supplemental Digital Content, https://links.lww.com/MD/P659). The quality assessment was independently reviewed by 2 authors (VMP and VSF). Inter-reviewer reliability was high, and any doubts or discrepancies were resolved through consultation with JMCT and JALA until a consensus was reached.

### 2.6. Data extraction

Data extraction was performed by VMP and VSF. The data extracted from each study included: (1) title, authors, and year; (2) study design; (3) population characteristics: sample size, age, sex, and selection; (4) study intervention; (5) main results; and (6) conclusions.

When interpreting the results, the intervention was considered effective if it increased the survival of OP with sepsis in the ED and adhered to the recommendations regarding ED response times, and the use of scales, protocols and biomarkers in the diagnosis.

### 2.7. Analysis of the data obtained

A narrative synthesis of the selected studies was conducted. An analysis was performed on the information provided about the situation of adult OP with infectious complications who presented to the ED, particularly regarding the times of care and the antibiotic treatments they received. Data on the impact of implementing protocols and using scales to improve the care provided to this group were also evaluated.

For quantitative synthesis, a meta-analysis was conducted using RevMan 5.4 software. This meta-analysis assessed the difference in the mean and standard deviation of antibiotic administration times between patients treated under a protocol in the ED and those who were not. Due to the heterogeneity in the remaining outcome variables, no further meta-analyses could be conducted. Heterogeneity was evaluated using the I^2^ value, with percentages ≤ 25%, 26% to 50%, and ≥ 51% indicating low, medium or high heterogeneity, respectively. Risk of publication bias was estimated by visual inspection of the funnel plot, and statistical significance was set at .05.

## 3. Results

### 3.1. Study selection

Through searches in various databases, a total of 1040 results were collected. After removing 206 duplicates, 834 results remained. By reading the titles and abstracts of these studies, 802 were excluded, leaving 32 articles for full-text review. Of these articles, 16^[4–6,16,20–22,24,26,34–40]^ were selected in the qualitative synthesis, and 5^[6,22,36,37,40]^ were used in the quantitative synthesis. The study selection process is illustrated in Figure 1.

### 3.2. Study characteristics

The included studies^[4–6,16,20–22,24,26,34–40]^ featured heterogeneous patient characteristics, as the review did not focus on a single type of cancer but rather addressed oncological pathology in general. The total number of participants across the studies was 8938, all of whom had oncological pathology and attended the ED due to febrile neutropenia (FN), sepsis or septic shock. Among the participants, 47.7% were females and 52.3% were males, all of whom were adults. Table 3 shows the characteristics of the included studies.

**Table 3:** Main results of the included studies.

Authors/year/country	Design	Participants	Intervention	Results	Conclusions	Risk of bias
Chen et al^[16]^ (2022) China	Retrospective cohort study	176 OP who came to the ED for septic shock. 141 patients with ST, and 35 patients with HT.	Examine the distribution of infections in OP and analyze the correlation between the infection site and mortality.	Overall mortality of 47.73%, with 78.26% due to gastrointestinal infections, followed by respiratory focus at 62.12% and urinary focus at 31.91% with *P*-value .002.	There is a relationship between the infection site and mortality. Gastrointestinal infections are associated with higher mortality rates.	10/11
Keng et al^[40]^ (2015) United States	Prospective cohort study	411 OP attended in the ED. 223 following a febrile neutropenia protocol (FNP), 87 historic cohort, and 101 receiving direct attention in the oncology department.	Analyze whether the implementation of FNP reduced the time to first attention in the ED.	57% of FNP patients received first dose of antibiotic within 90 min, 13% in the historic cohort and 1% in patients receiving direct attention, *P*-value < .05. The time to first physician attention was 43 min for FNP, 73 min for the historic cohort, and 20 min for direct attention, *P*-value < .001.	The application of FNP significantly reduced the time to first attention for OP attending the ED.	10/11
Morneau et al^[39]^ (2017) United States	Retrospective observational study	100 OP with sepsis attended in the ED.	Evaluate the association between in-hospital mortality and time to antibiotic administration in septic OP.	The median time to antibiotic administration was 3.4h for survivors vs 9.8 h for non-survivors, *P*-value = .17. The in-hospital mortality OR = 1.16 per hour delay in antibiotic administration, *P*-value = .04.	Timely antibiotic administration is crucial in reducing in-hospital mortality for OP with sepsis.	9/11
Bader et al^[22]^ (2020) Jordan	Quasi-experimental study	168 subjects with oncological pathology who attended the ED with suspected sepsis.	A protocol based on qSOFA scores ≥ 2 was applied. The protocol activities included: (1) nursing order, (2) laboratory, (3) radiology, (4) medication, and (5) ICU consultation.	The efficacy of the protocol was evaluated based on time reduction: (A) from 20 to 6 min. Triage – MA. (B) from 44 to 20 min. Triage – analysis. (C) from 53 to 20 min. Triage – fluid resuscitation. (D) from 95 to 45 min. Triage – antibiotic. Mortality was reduced by 11.7% following the implementation of the protocol.	The implementation of the protocol significantly reduced assistance times. Nursing personnel play a critical role in the early detection of sepsis using qSOFA during triage.	9/9
Mattison et al^[38]^ 2016 United Kingdom	Retrospective observational study	697 OP with sepsis attended in the ED.	Analyze the association between a nurse-led protocol based on Patient Group Directions (PGD) and the time to first antibiotic administration.	48.1% of patients received their first antibiotic dose within 15 min, 17.6% within 30 min, 20.5% within 45 min, 13.8% within 60 min, and 3.6% in more than 60 min.	Nurse-led protocols improved the rates at which septic OP received timely antibiotic doses.	11/11
Koh et al^[21]^ (2020) Australia	Retrospective cohort study	1655 OP who came to the ED. Cohorts divided depending on qSOFA score < 2 (n = 1284) or ≥ 2 (n = 371)	Evaluate the performance of qSOFA as a predictor of mortality and ICU stay ≥ 3 d.	Mortality or ICU stay ≥ 3 d with qSOFA ˂ 2 was 6.9% vs qSOFA ≥ 2 was 21% with *P*-value < .001. More than 50% of the deceased had qSOFA ≥ 2, while 80% of the survivors obtained qSOFA ˂ 2.	Scores in qSOFA ≥ 2 are associated with a 3-fold increase in mortality.	10/11
Hanzelka et al^[37]^ (2013) United States	Retrospective cohort study	200 septic OP attended in the ED. 100 patients attended before vs 100 patients attended after the implementation of an early-goal directed therapy (EGDT) protocol.	Implementation of EGDT protocol to improve the care offered to OP with sepsis in the ED.	Mortality rates were 38% before vs 20% after EGDT implementation, *P*-value = .005. Mean hospital length of stay was 10.3 ± 11.6 d before vs 8.1 ± 6 d after, *P*-value = .160. The mean time to lactate determination was 195 ± 407 min before vs 120 ± 307 min after, *P*-value = .147.	The EGDT protocol was associated with lower mortality rates and improved the quality of care offered in the ED.	10/11
Peyrony et al^[20]^ (2020) France	Prospective observational study	249 OP with FN who came to the ED.	Investigate the following predictors of mortality and ICU admission: (1) antibiotic initiation time, (2) antibiotic group used, (3) high shock index, and (4) low performance status.	Patients admitted to ICU or who died had an antibiotic start time of 66 min vs 92 min (*P* = .097). Factors associated with ICU admission or death were: (1) inappropriate use of antibiotics (aOR = 3.50, *P* = .004;), (2) high shock index (≥1), (aOR = 2.53, *P* = .02) and (3) low performance state (˃2) (aOR = 8.89, p ˂ .00001).	An inadequate antibiotic regimen and high dependency levels were associated with higher ICU admissions, high shock indices, and death. No association was found between the time to start antibiotic therapy and poor prognosis.	11/11
Lee et al^[36]^ (2018) Korea	Retrospective cohort study	104 OP with FN presented to the ED. 25 cases either died or were admitted in ICU vs 79 controls who were discharged from the ED.	Identify prognostic factors predicting poor outcomes in OP with FN attending the ED.	The qSOFA score was 0.88 ± 0.78 in cases group vs 0.36 ± 0.53 in control group, *P*-value < .001. The aOR of poor outcomes was 29.65, 95% CI (3.81–230.7) for respiratory tract infections, *P*-value = .0012.	The qSOFA score proved useful for assessing OP with FN in the ED. Elevated qSOFA scores and respiratory tract infections were associated with worse prognoses.	11/11
Jung et al^[5]^ (2020) South Korea	Retrospective observational study	133 OP with neutropenic septic shock secondary to chemotherapy attending the ED	Investigate infectious agents and bacterial resistances among OP attending the ED.	77.1% of infections were gram-negative, primarily gastrointestinal. The most common MDR organism was ESBL-producing *E. coli* (50%). 48% and 72.2% of the MDR were resistant to beta-lactams such as cefepime and ceftazidime.	Gram-negative bacteria were the most common infectious agents. MDR organisms were resistant to beta-lactam monotherapy, but were sensitive to piperacillin-tazobactam or carbapenem.	9/11
Chaftari et al^[4]^ (2021) United states	Retrospective observational study	3623 OP presenting to the ED with suspected infection.	Combine the qSOFA score, cancer type and 5 biomarkers to assess mortality risk in 4 groups according to cancer type.	14-d mortality rates were 0.4% vs 4.2% vs 14.2% vs 41.2% for low, intermediate, high and very high risk groups, *P*-value < .001. Increases in qSOFA scores were positively correlated with increases in biomarkers (*P* < .001).	Certain markers such as CRP, lactate, and procalcitonin are independent predictors of short-term mortality and ICU admission but should be used in conjunction with other diagnostic tests.	10/11
Alsharawneh et al^[24]^ (2020) Canada	Retrospective observational study	431 OP presenting to the ED with fever	Evaluate the quality of care received, considering the safety, effectiveness and timeliness of the IDSA guidelines.	36% of patients were discharged, and 11% were treated within the time proposed by the guidelines. The mean time from triage to antibiotic administration was 228 min. The classification time was 5.4 h, boarding time was 10.6 h, and ED stay time was 21.9 h with *P*-value < .001.	Regarding safety and time, none of the proposed domains met the guidelines. In terms of effectiveness, the data suggests that most patients received inappropriate triage.	10/11
Sheward et al^[26]^ (2022) England	Quasi-experimental study	70 patients with suspected neutropenic sepsis according to NEWS-2 ≥ 5 or 1 item ≥ 3 points	Implement Advanced Clinical Practitioners (ACP) trainers and a holistic screening system with a decision-making algorithm.	Before the intervention, 17% of patients received antibiotics. After the intervention, 100% of the patients received IV antibiotics within the first hour. The usage of PGD increased from 30% to 93%.	The introduction of ACP has significantly improved the care and prognosis of patients with neutropenic sepsis.	8/9
André et al^[35]^ (2010) France	Prospective cohort study	197 OP who came to the ED. 89 OP with severe sepsis and 108 with non-severe sepsis.	Evaluate the management of septic OP in the ED and discuss whether that management complies with recommendations.	In the total sample, 83% of patients received inadequate care. In the severe sepsis group, 22% of subjects received antibiotics within the first 90 min vs 84% of patients with non-severe sepsis.	The management in ED does not comply with recommendations for OP with sepsis attending the ED.	10/11
Alsharawneh^[6]^ (2021) Jordan	Retrospective cohort study	423 OP admitted with a diagnosis of FN (n = 79), sepsis (n = 214), septic shock (n = 39), and comparison cohort (n = 91)	Study the impact of triaging with a lower priority level than recommended for various groups compared to a comparative cohort.	The hours of medical care for the groups (NF vs sepsis vs septic shock vs control) were 56.9 vs 70.5 vs 91.5 vs 83.8 (*P* < .001). The antibiotic administration times were 38.6 vs 81.2 vs 94.9 vs 101.5 min (*P* < .001). 96.3% of sepsis patients and 86.8% of controls were under-triaged.	It is the responsibility of the triage nursing staff to accurately and promptly identify these patients.	10/11
Chae et al^[34]^ (2020) South korea	Retrospective cohort study	301 septic OP presenting to the ED. Cohorts divided on survivals (n = 258) and non-survivals (n = 43) in 30 d.	Evaluate the prognostic accuracy of the SOFA and qSOFA scales for mortality.	For the SOFA scale, 30-d mortality was 23.2% for scores ≥ 2 vs 3% for scores < 2 (*P* < .001). The qSOFA scale showed a 30-d mortality of 47.8% for scores ≥ 2 vs 11.5% for scores < 2 (*P* < .001).	Higher scores on the SOFA and qSOFA scales are associated with worse prognosis and higher 30-d mortality in OP.	10/11

ACP = advanced clinical practitioners, aOR = adjusted odds ratio, ED = emergency department, EGDT = early-goal directed therapy, ESBL = extended spectrum beta-lactamase, FN = febrile neutropenia, FNP = febrile neutropenia protocol, HT = hematological tumor, ICU = intensive care unit, MA = medical attention, IDSA = infectious diseases society of america, MASCC = Multinational Association for Supportive Care in Cancer, MASCC score <21 = high risk of FN, MASCC score ≥21 = low risk of FN, MDR = multidrug-resistant bacteria, NEWS-2 = National Early Warning Score 2, NEWS-2 score <5 = low risk of deterioration, NEWS-2 score ≥5 = high risk of deterioration and subsequent complications, OP = oncologic patient, PGD = patient group directions, qSOFA = quick sequential organ failure assessment, qSOFA score <2 = low risk of deterioration, qSOFA score ≥2 = high risk of mortality, ST = solid tumor.

### 3.3. Risk of bias

A bias assessment was conducted on the 16 studies^[4–6,16,20–22,24,26,34–40]^ included in the narrative synthesis. For this purpose, the JBI tools for observational cohort studies (Table S1, Supplemental Digital Content, https://links.lww.com/MD/P659) and quasi-experimental studies (Table S2, Supplemental Digital Content, https://links.lww.com/MD/P659) were used.^[33]^

Regarding the evaluation of the cohort studies, it is noteworthy that all studies received high scores when applying this tool, with figures ranging from 9 to 11 points out of a total of 11. Additionally, concerning the quality of the quasi-experimental studies, the scores were equally high, ranging from 8 to 9 points out of a total of 9.

### 3.4. Infectious patterns in cancer patients with sepsis

The presence of infections in OP is more severe than in the rest of the population attending the ED. The spectrum of infectious agents is broad, but it is estimated that the main microorganisms causing bacteremia are gram-negative and that their origin is respiratory.^[5,16,20]^ The infection sites with the highest mortality rates are gastrointestinal and respiratory,^[16,36]^ while genitourinary infections are associated with a lower risk of death.^[16]^ It is hypothesized that infections of unknown origin could be more severe, as it is impossible to identify the causative agent and, consequently, specific treatment cannot be administered.^[16]^

30.3% of causal pathogens are MDR bacteria, with ESBL-producing *E. coli* being the most common (17.5%).^[5]^ The specific resistance patterns of each bacterial strain will determine the appropriate treatment.^[5]^ Furthermore, the use of an inadequate antibiotic regimen is associated with a worse prognosis.^[20]^ It is estimated that 48% of MDR bacteria possess resistance to beta-lactam monotherapy.^[5]^ However, they are the most widely used group of antibiotics in routine clinical practice.^[20]^ On the other hand, resistance to antibiotics such as carbapenem or piperacillin/tazobactam appears to be nonexistent, so their use is recommended. Additionally, in case of complications, the administration of aminoglycosides would be indicated.^[5,20]^

### 3.5. Emergency care for cancer patients with sepsis

The care provided in ED generally does not comply with international recommendations.^[6,22,24,26,35,36,39,40]^ Waiting times within the service significantly exceed the 60-minute limit, with time to antibiotic administration extending to 81 minutes and 3.4 hours.^[6,22,24,26,35,36,39,40]^ Additionally, only 13.8% to 22% of patients receive timely antibiotic treatment when a protocol is not implemented.^[26,36,38]^

The triage system plays a key role in the origin of this problem.^[6,22,24,39]^ A large proportion of patients experience delays in triage, consequently increasing overall care times, particularly the time to initiate antibiotics,^[6,39]^ although some authors discuss this idea.^[20]^ Even when patients are triaged with priority level 2 (requiring initial medical attention within 15 minutes), some are not treated within the recommended timeframe.^[6]^ Notably, the proportion varies depending on the pathology (70% for patients with FN and 26.7% for patients with sepsis).^[6,24]^ Furthermore, even in cases where action protocols are established, the professionals working in the ED often lack sufficient training, leading to triage errors, insecurity, and a lack of determination to act.^[6,26]^

### 3.6. Use of scales and implementation of protocols

Implementing protocols has proven to have significant benefits.^[4,22,26]^ The incorporation of ACP improved OP care and prognosis.^[26]^ Before the implementation of ACP, 17% of patients visiting the ED received the first dose of antibiotic in the first hour, whereas after the intervention, this figure rose to 83%.^[26]^ Similarly, developing action protocols based on the implementation of scales and categories of activities for the multidisciplinary team, reduced the time between triage and the first dose of antibiotic from 95 to 45 minutes postintervention.^[22]^ Additionally, a protocol that uses scales in conjunction with 5 biomarkers to classify ED patients based on the risk of mortality and ICU admission revealed that the highest risks of 14-day mortality corresponded to subjects with elevated levels of procalcitonin, lactate, lactate dehydrogenase, and albumin with odds ratios (OR) of 2.35 ± 0.399, 3.17 ± 0.234, 3.65 ± 0.245 and 3.08 ± 0.287, all statistically significant.^[4]^ Systematization and objectivity in care reduce professional bias and allow for quicker, more effective action in septic complications.^[4,22,26]^

**Figure 2.**
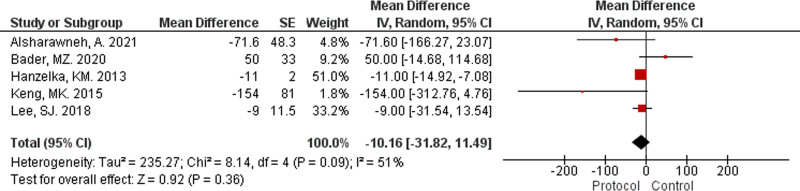
Protocol impact on antibiotic time.

**Figure 3.**
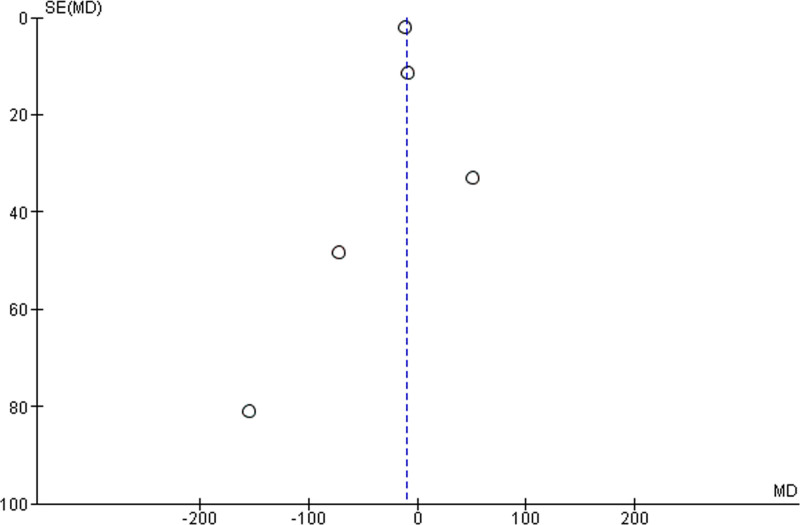
Publication bias for protocol implementation.

Following the implementation of protocols, antibiotic administration times improved.^[6,22,36,37,40]^ This is demonstrated in a random-effects meta-analysis (Fig. 2), which showed that the application of protocols reduced the mean antibiotic administration times by −10.16 ± 21.65 minutes with an I^2^ heterogeneity of 51%. The risk of publication bias was assessed through visual inspection of the funnel plot (Fig. 3).

Regarding the use of scales, they should be used with caution.^[4,20–22,34,36]^ The mortality or ICU stay for patients with a qSOFA score ≥ 2 is 21% vs 6.9% for those with scores less than 2, *P*-value < .001.^[21]^ Similarly, another study^[34]^ found that scores on the SOFA and qSOFA scale ≥ 2 were related to higher 30-day mortality rates, with *P*-value < .001 and an area under the curve of 0.79 ± 0.08 for the SOFA scale and 0.66 ± 0.1 for the qSOFA scale. Additionally, the time between triage and antibiotic administration was shorter in patients with qSOFA scores ≥ 2 compared to those with scores lower than 2 (3.3 vs 2.8, *P*-value < .001); however, this study found lower sensitivity of the scale compared to non-oncological patients (52% vs 61%), but without statistical significance.^[21]^ Similarly, a study^[22]^ that used the qSOFA as a triage tool as part of his protocol found an improvement in the percentage of subjects receiving antibiotics within the first hour from 10.8% to 89.4% (*P* < .001), and decreased mortality by 11.7% once the scale was applied.^[22]^

High scores on the qSOFA scale were associated with an OR of 14-day mortality of 9.23 ± 0.249 (*P*-value < .001)^[4]^; however, a very high percentage of the deceased individuals (83.5%) had a qSOFA score of less than 2. Additionally, another study found an OR for poor outcomes in OP of 4.62 ± 3.45 (*P*-value = .285) in subjects with high qSOFA scores.^[36]^ As with the scale, the use of biomarkers alone is not sufficiently accurate for diagnosis, but they are useful for guiding decision-making.^[4,36]^

Another study^[20]^ based on the internationally recommended MASCC risk index to stratify its sample according to the risk of complications found that applying this scale presented a sensitivity and specificity of 0.78 and 0.43%, respectively, with an area under the ROC curve of 0.761 for predicting the absence of complications. This risk index was able to classify high- and low-risk patients with certainty, predicting fewer complications in low-risk patients than in high-risk patients (32.4% and 56.6%, *P*-value = .0006). However, it could be imprecise in discerning low-risk patients in the ED), especially if they are oncohematological, as 16% of these patients’ presented complications.

## 4. Discussion

Based on the results obtained, the requirement for immediate treatment in these patients is generally not met, as waiting times exceed the recommended maximums.^[6,22,24,26,35,36,39,40]^ Therefore, it is necessary to implement changes to improve the system’s functioning, such as implementing protocols and using scales.^[6,20,22,24,26]^ According to international recommendations from IDSA, NICE or Surviving Sepsis Campaign, septic complications require immediate treatment (within the first hour) with broad-spectrum antibiotics, as they are time-dependent pathologies.^[6,22,24,26,27]^ These recommendations are consistent with the results of a Randomized Clinical Trial (RCT) that demonstrated how shorter initial care times improved the 28-days mortality patients with sepsis treated in the ED.^[41]^

However, in the present review, one study^[20]^ differs regarding the need for immediate antibiotics, as it did not find a correlation between the time of administration of the first antibiotic dose and prognosis, although this result was not significant (*P*-value = .7). These results could be because a large part of their sample (69.5%) had a low risk of complications according to MASCC, and approximately 90% had a good performance status. Notably, both IDSA and EUSEM show caution regarding the recommendations published in the latest Surviving Sepsis Campaign,^[28]^ as the massive administration of broad-spectrum antibiotics to patients could increase bacterial resistance; despite this, they conclude by recommending following the guidelines and adhering to that time limit.^[20]^

The studies in this review concur with the literature that the empirical selection of antibiotics should be performed in collaboration with microbiologists, considering epidemiological patterns and local bacterial resistance.^[5,25,27,29–31]^ Monotherapy with broad-spectrum beta-lactams, mainly piperacillin/tazobactam or carbapenem, is recommended.^[12,25,27,29,31]^ In severe cases, a second antibiotic (aminoglycoside) would be administered.^[12,25,27,29,31]^ This approach is supported by several authors^[5,20]^; however, one study^[20]^ suggests that dual therapy (piperacillin/tazobactam along with aminoglycoside) should be initiated from the beginning. This aligns with findings from other authors^[12]^ who reported that penicillins and cephalosporins are the second most used antibiotics (88.8%) in patients with sepsis and septic shock.^[12]^ Only glycopeptides are more commonly used, and most pathogens isolated in these patients are gram-negative and sensitive to this antibiotic.^[4,5,15,29]^ Despite this, coverage against gram-positive bacteria is also recommended if hemodynamic instability or signs of severe sepsis develop.^[12]^ This could be justified as pneumonia, similar to the results of the present review, is the most frequent infectious focus in OP^,[5,16,20,36]^ and the use of glycopeptides such as vancomycin would be indicated, as demonstrated by this review in accordance with the current literature.^[7,10,14,15]^

Currently, there is no specific diagnostic tool available, but numerous tests and tools are employed to guide diagnosis, among which the scales used in studies presented in this work stand out: qSOFA and MASCC.^[4,20–22,34,36]^ The authors of these studies agree with the general idea that these scales should not be used solely as diagnostic instruments, but rather as classification tools to guide decision-making regarding patient management based on their severity or risk of deterioration.^[4,6,14,20–22,24,25,27,29,30,34,36]^

The qSOFA scale is recommended by guidelines such as Sepsis-3 or Surviving Sepsis Campaign,^[22,28,29]^ and has demonstrated great utility as a complementary tool in this review.^[4,21,22,34,36]^ This finding aligns with a previous study^[42]^ that reported the qSOFA scale’s usefulness in predicting complications and 28-day mortality in patients with sepsis. However, one study^[4]^ reported unfavorable results in predicting mortality in OP using the qSOFA scale, possibly due to the lack of information such as the type of cancer. The guidelines suggest caution with the use of qSOFA and prefer other scales (NEWS, SIRS, and MEWS) in OP because, as other authors point out, it could be less sensitive due to the alterations caused by the disease itself and its treatments.^[21,25,28]^ This is consistent with the results of Sheward et al^[26]^ who used the NEWS scale and obtained favorable results, similar to those seen in non-oncological patients to predict the risk of complications from gram-negative infections.^[43]^ However, it should be noted that, although the qSOFA or NEWS scales are optimal, there is no defined international scale for this type of patient.

On the other hand, the MASCC risk index is equally recommended by guidelines such as Sepsis-3 and NICE 2012.^[27]^ Its use allows stratifying patients according to risk, guiding their management. Those at low risk could be treated with oral antibiotics at home without the need for hospitalization.^[5,24,30]^ This recommendation aligns with the results of a study included in the present review^[24]^ and other publications that reported beneficial outcomes when implementing the MASCC scale.^[44,45]^ Conversely, Peyrony et al^[20]^ highlighted that MASCC could be less accurate in detecting patients with FN at low risk of complications; this may be because the OP who visit the ED are in worse health than those already hospitalized or free of oncological processes.^[6,20]^

The ED is typically the first point of care for OP with septic complications.^[4]^ However, the care provided in these services does not meet the minimum standards in terms of action times and objectives, as highlighted in this review.^[6,20,22,24,26,35–40]^ The delay in initial management is critical, as septic complications are time-dependent.^[4–6,22,24,26,27,36–40]^ In this group, the progression of the condition can be fulminant.^[21,24,29]^ Despite this, many patients go unnoticed since OP can present atypical symptoms that mask the infection.^[14,27]^ This finding aligns with the results of other studies,^[6,46]^ which highlighted problems mainly in triage. Additionally, the lack of personnel and excessive workload, along with waiting for a medical prescription, exacerbate the situation.^[27]^

In response to the need for immediate attention to these patients, certain authors^[4,37]^ have opted to combine different sources of information (scales, analytical biomarkers and types of cancer) in their predictor protocol. This aligns with other publications,^[22,26]^ which managed to increase the percentage of patients who met the recommended care times, reduce the mortality rate, implement ACP, and create an action protocol.

Institutions such as the National Confidential Inquiry into Patient Outcome and Death (NCEPOD), National Chemotherapy Advisory Group (NCAG) and NICE-2020 recommend updating hospital ED by incorporating guidelines and action protocols such as “Sepsis Six” and a service of acute oncological emergencies specializing in oncological complications such as neutropenic sepsis.^[27]^ These recommendations align with the results obtained by Sheward et al^[26]^ who significantly improved ED care by incorporating ACP, and the results of previous reviews^[47–49]^ that highlight the importance of this approach in the context of care.

### 4.1. Limitations and strengths

This review focuses on providing a general description of the management of septic complications in the OP. Given the heterogeneity of cancer, it is likely that the recommendations could vary depending on the type and the stage of the disease. Similarly, the specific management of each type of infection, which could vary, has not been described. Furthermore, the information used comes from descriptive studies that do not present a level of evidence as high as RCT; therefore, the information presented may not be generalizable in all cases.

Regarding strengths, a meta-analysis was performed assessing the impact on time to antibiotics when a protocol was applied. Additionally, studies conducted in various regions of the world with a sample of almost 9.000 patients in total have been included, with similar results obtained in most studies. Notably, several of these studies applied internationally validated scales for the assessment of OP.^[4,20–22,26,35,36,40]^

### 4.2. Implications for clinical practice

With cancer cases increasing annually and sepsis/septic shock being leading causes of morbidity and mortality among this group,^[14]^ healthcare personnel must be prepared to attend to an increasing number of OP with complications in the ED, including septic complications.^[26]^ Nursing is in a unique position to detect system failures and lead changes in the organization and functioning of these services.^[22,24,38]^ Nurses are responsible for triage and must therefore be able to accurately identify infectious complications in OP.^[6]^ The role of ACP has been shown to significantly improve this issue,^[26,47–49]^ so its implementation in various hospital services, especially in the ED, should be considered.

The training of healthcare professionals, especially triage nurses, should be promoted so that they can detect these patients early and act accordingly.^[6,14,27]^ For this purpose, they must know the typical and atypical manifestations of infectious processes in OP.^[14,16]^ Similarly, the training of nursing professionals helps to improve confidence and proactivity when acting and using protocols in the ED.^[26]^

The multidisciplinary perspective has great benefits for hospital services; however, enhancing the independence of nursing staff through the use of protocols for the administration of antibiotics exempt from medical orders, such as the PGD in the United Kingdom, or focusing attention on the implementation of protocols led by this group, could accelerate the patient care process and the administration of the first dose of antibiotics, thus improving patient outcomes.^[26,38]^

## 5. Conclusion

OP have a higher likelihood of infectious complications and exhibit higher mortality rates. With an increasing number of OP experiencing immunosuppressive sequelae, healthcare professionals must identify the needs of this group and act accordingly. Emergency care generally does not meet minimum quality standards, especially regarding waiting times. Therefore, it is essential to improve the functioning of these services by employing systematic and multidisciplinary protocols along with the use of predictive scales. In this regard, nursing professionals play an indispensable role in this process, as their position in the ED facilitates the early detection of these complications. Training and enhancing the independence of nurses are key to streamlining the emergency care process.

Protocols used in the ED should include various tests to guide management and decision-making, highlighting immediate-use scales such as the MASCC index or qSOFA, which demonstrate greater accuracy when used with other clinical and laboratory findings. Additionally, the use of protocols showed a decease in time to antibiotics, which may contribute to enhance outcomes in OP with sepsis. A synthesis of the literature on the subject has been conducted; however, the lack of RCT necessitates conducting such studies, focusing on specific types of cancer and considering the stage of the disease as well as the type of infection the patient is experiencing. Future trials should clarify the efficacy of nurse-led protocols and the use of scales among this population, creating new evidence to guide clinical practice in the ED.

## Author contributions

**Conceptualization:** Victor Melgar-Perez, Victor Serrano-Fernandez, Joseba Aingerun Rabanales-Sotos, Jose Alberto Laredo-Aguilera.

**Data curation:** Victor Melgar-Perez, Victor Serrano-Fernandez, Joseba Aingerun Rabanales-Sotos, Jose Alberto Laredo-Aguilera.

**Formal analysis:** Victor Melgar-Perez, Victor Serrano-Fernandez, Carmen Bouzas-Mosquera, Jose Alberto Laredo-Aguilera.

**Funding acquisition:** Victor Serrano-Fernandez, Juan Manuel Carmona-Torres, Carmen Bouzas-Mosquera, Joseba Aingerun Rabanales-Sotos, Jose Alberto Laredo-Aguilera.

**Investigation:** Victor Melgar-Perez, Victor Serrano-Fernandez, Carmen Bouzas-Mosquera, Joseba Aingerun Rabanales-Sotos, Jose Alberto Laredo-Aguilera.

**Methodology:** Victor Melgar-Perez, Victor Serrano-Fernandez, Juan Manuel Carmona-Torres, Carmen Bouzas-Mosquera, Jose Alberto Laredo-Aguilera.

**Project administration:** Juan Manuel Carmona-Torres, Carmen Bouzas-Mosquera.

**Resources:** Juan Manuel Carmona-Torres.

**Software:** Victor Serrano-Fernandez, Juan Manuel Carmona-Torres.

**Supervision:** Jose Alberto Laredo-Aguilera.

**Validation:** Juan Manuel Carmona-Torres.

**Visualization:** Victor Serrano-Fernandez, Joseba Aingerun Rabanales-Sotos, Jose Alberto Laredo-Aguilera.

**Writing – original draft:** Victor Melgar-Perez, Victor Serrano-Fernandez, Joseba Aingerun Rabanales-Sotos, Jose Alberto Laredo-Aguilera.

**Writing – review & editing:** Victor Melgar-Perez, Victor Serrano-Fernandez, Juan Manuel Carmona-Torres, Carmen Bouzas-Mosquera, Joseba Aingerun Rabanales-Sotos, Jose Alberto Laredo-Aguilera.

## Supplementary Material


