# Supplementary material for: Initial management of septic complications in adult oncologic patients in the emergency department: A systematic review and meta-analysis
Source: Medicine (Baltimore). 2025 Nov 28;104(48):e43804. doi: 10.1097/MD.0000000000043804 (PMC12662436; doi:10.1097/MD.0000000000043804)
Supplement: Supplementary file 1 [file medi-104-e43804-s001.docx]

Supplementary Table 1. JBI score for Observational Cohort studies

|  | **Item 1** | **Item 2** | **Item 3** | **Item 4** | **Item 5** | **Item 6** | **Item 7** | **Item 8** | **Item 9** | **Item 10** | **Item 11** | **Overall score** |
| --- | --- | --- | --- | --- | --- | --- | --- | --- | --- | --- | --- | --- |
| **Chen Y, et al.**  **2022** | 1 | 1 | 1 | 1 | 1 | 1 | 1 | 1 | 1 | 0 | 1 | 10/11 |
| **Keng M, et al.**  **2015** | 1 | 1 | 1 | 1 | 1 | 1 | 0 | 1 | 1 | 1 | 1 | 10/11 |
| **Koh TL, et al.**  **2021** | 1 | 1 | 1 | 1 | 1 | 1 | 1 | 1 | 1 | 0 | 1 | 10/11 |
| **Peyrony O, et al. 2020** | 1 | 1 | 1 | 1 | 1 | 1 | 1 | 1 | 1 | 1 | 1 | 11/11 |
| **Morneau K, et al.**  **2017** | 1 | 1 | 1 | 0 | 0 | 1 | 1 | 1 | 1 | 1 | 1 | 9/11 |
| **Mattison G, et al.**  **2016** | 1 | 1 | 1 | 1 | 1 | 1 | 1 | 1 | 1 | 1 | 1 | 11/11 |
| **Jung S, et al.**  **2020** | 1 | 1 | 1 | 1 | 0 | 1 | 1 | 1 | 1 | 0 | 1 | 9/11 |
| **Chaftari P, et al.**  **2021** | 1 | 1 | 1 | 1 | 1 | 1 | 1 | 1 | 1 | 0 | 1 | 10/11 |
| **Hanzelka KM, et al.**  **2013** | 1 | 1 | 1 | 1 | 1 | 1 | 1 | 1 | 1 | 0 | 1 | 10/11 |
| **Lee, S, et al.**  **2018** | 1 | 1 | 1 | 1 | 1 | 1 | 1 | 1 | 1 | 1 | 1 | 11/11 |
| **Alsharawneh A, et al. 2020** | 1 | 1 | 1 | 1 | 1 | 1 | 1 | 1 | 1 | 0 | 1 | 10/11 |
| **Alsharawneh A, et al. 2021** | 1 | 1 | 1 | 1 | 1 | 1 | 1 | 1 | 1 | 0 | 1 | 10/11 |
| **André S, et al.**  **2010** | 1 | 1 | 1 | 1 | 0 | 1 | 1 | 1 | 1 | 1 | 1 | 10/11 |
| **Chae, B, et al.**  **2020** | 1 | 1 | 1 | 1 | 0 | 1 | 1 | 1 | 1 | 1 | 1 | 10/11 |

Supplementary Table 2. JBI score for Quasi-Experimental studies

|  | **Item 1** | **Item 2** | **Item 3** | **Item 4** | **Item 5** | **Item 6** | **Item 7** | **Item 8** | **Item 9** | **Overall score** |
| --- | --- | --- | --- | --- | --- | --- | --- | --- | --- | --- |
| **Bader M, et al. 2020** | 1 | 1 | 1 | 1 | 1 | 1 | 1 | 1 | 1 | 9/9 |
| **Sheward N, et al. 2022** | 1 | 0 | 1 | 1 | 1 | 1 | 1 | 1 | 1 | 8/9 |
